# Differential expression of m^5^C RNA methyltransferase genes *NSUN6* and *NSUN7* in Alzheimer’s disease and traumatic brain injury

**DOI:** 10.1007/s12035-022-03195-6

**Published:** 2023-01-17

**Authors:** Adriana PerezGrovas-Saltijeral, Anto P. Rajkumar, Helen Miranda Knight

**Affiliations:** 1 Division of Cells, Organisms and Molecular Genetics, School of Life Sciences, University of Nottingham, Nottingham, UK; 2 Institute of Mental Health, Mental Health and Clinical Neurosciences Academic Unit, School of Medicine, University of Nottingham, Nottingham, UK; 3 Mental Health Services for Older People, Nottinghamshire Healthcare NHS Foundation Trust, Nottingham, UK

**Keywords:** Alzheimer’s disease, 5-Methylcytosine methylation, NOL1/NOP2/sun domain family genes, NSUN6, Traumatic brain injury

## Abstract

Epigenetic processes have become increasingly relevant in understanding disease-modifying mechanisms. 5-Methylcytosine methylations of DNA (5mC) and RNA (m^5^C) have functional transcriptional and RNA translational consequences and are tightly regulated by writer, reader and eraser effector proteins. To investigate the involvement of 5mC/5hmC and m^5^C effector proteins contributing to the development of dementia neuropathology, RNA sequencing data of 31 effector proteins across four brain regions was examined in 56 aged non-affected and 51 Alzheimer’s disease (AD) individuals obtained from the Aging, Dementia and Traumatic Brain Injury Study. Gene expression profiles were compared between AD and controls, between neuropathological Braak and CERAD scores and in individuals with a history of traumatic brain injury (TBI). We found an increase in the DNA methylation writers *DNMT1*, *DNMT3A* and *DNMT3B* messenger RNA (mRNA) and a decrease in the reader *UHRF1* mRNA in AD samples across three brain regions whilst the DNA erasers *GADD45B* and *AICDA* showed changes in mRNA abundance within neuropathological load groupings. RNA methylation writers *NSUN6* and *NSUN7* showed significant expression differences with AD and, along with the reader *ALYREF*, differences in expression for neuropathologic ranking. A history of TBI was associated with a significant increase in the DNA readers *ZBTB4* and *MeCP2* (*p* < 0.05) and a decrease in *NSUN6* (*p* < 0.001) mRNA. These findings implicate regulation of protein pathways disrupted in AD and TBI via multiple pre- and post-transcriptional mechanisms including potentially acting upon transfer RNAs, enhancer RNAs as well as nuclear-cytoplasmic shuttling and cytoplasmic translational control. The targeting of such processes provides new therapeutic avenues for neurodegenerative brain conditions.

**Graphical abstract:**

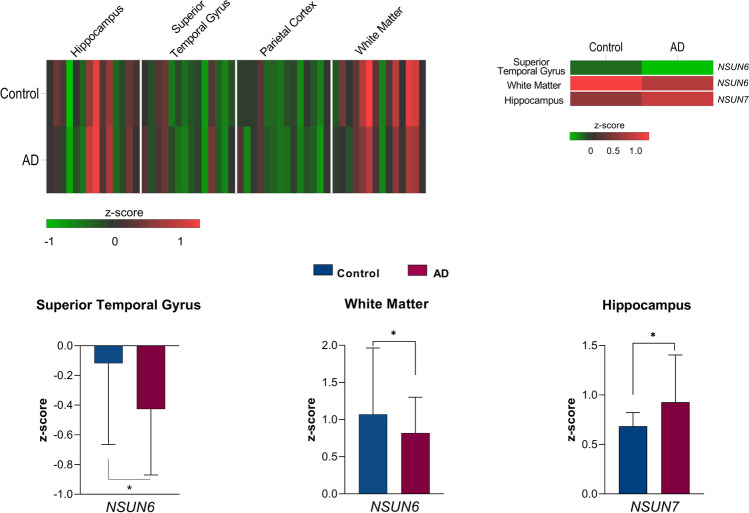

**Supplementary Information:**

The online version contains supplementary material available at 10.1007/s12035-022-03195-6.

## Introduction

Dementia, a progressive decline in neurocognitive ability, is the principal cause of disability in the elderly population [1]. In 2019, the number of people diagnosed with dementia worldwide reached 50 million, incurring a significant socio-economic burden on society [2, 3]. Forms of dementia are caused by progressive and irreversible degeneration of neurons and include Alzheimer’s disease (AD), dementia of Lewy bodies and frontotemporal dementia. Diagnosis of AD by brain tissue histology is made through the identification of intraneuronal aggregation of tau protein known as neurofibrillary tangles (NFTs) and the accumulation of insoluble beta-amyloid peptide (Aβ) termed senile and neuritic plaques [4]. These pathological features progress through stages of severity which correlate to burden of pathology and anatomical localization [5, 6].

Most neurodegenerative dementias have a multifactorial aetiology and are thought to develop due to multiple environmental and genomic factors. One proposed life event risk factor is a history of traumatic brain injury (TBI) which results from head trauma that leads to either focal or diffuse injury [7, 8]. The effect of TBI can be acute or chronic with the latter having a greater link to cognitive decline [9–11]. Neurodegenerative lesions are commonly found in individuals exposed to TBI and are referred to as TBI-related neurodegeneration (TReND) [10, 12, 13]. However, although changes in clinical phenotype, brain activity and histopathological features have been reported in TBI and TReND, less is known about the molecular mechanisms which contribute to long-term neuropathological changes.

Epigenetic processes which regulate transcriptional and translational activity are proposed as mechanisms which mediate environmental influences on brain physiology [14, 15]. Such epigenetic modifications are tissue specific and change during cellular maturation, i.e. during differentiation and ageing, and therefore have been implicated in disease processes including dementia pathology [16–18]. A commonly studied epigenetic process, 5-methylcytosine (5mC) DNA methylation, involves the addition of a methyl group to the fifth carbon of the cytosine base at CpG (mCG) and CpA (mCA) sites and is associated with transcription repression [19]. However, 5mC can be converted into alternative oxidised methylation states known as 5-hydroxymethylcytosine (5hmC), 5-formylcytosine (5fC) and 5-carboxylcytosine (5caC) through ten-eleven translocation (TET) methylcytosine dioxygenase and thymine DNA glycosylase (TDG)–mediated active demethylation [20, 21]. Such oxidised states have been shown to be stable and to potentially have functional transcriptional consequences [22–24].

Methylation of RNA species at the same cytosine base, known as 5-methylcytosine (m^5^C), also undergoes oxidisation to form 5-hydroxymethylcytosine (hm^5^C), 5-formylcytidine (f^5^C) and 5-carboxycytidine (ca^5^C) [25–28]. m^5^C methylation of messenger RNA (mRNA) is indicated to increase RNA stability and abundance and regulate nuclear exportation, negatively correlating with translation efficiency [29, 30]. In transfer RNA (tRNA), ribosomal RNA (rRNA) and mt-tRNA, m^5^C modification provides structural stability and improves the accuracy of translation [28, 29]. Similarly, hm^5^C modification enhances mRNA translation whereas f^5^C of mitochondrial tRNA is important for translation of AUA to methionine [31–33]. As such, the modification of DNA and RNA may have important consequences on cellular localisation of RNAs and regional patterns of gene expression.

DNA and RNA methylation are governed by three families of effector proteins known as writers, readers and erasers. The writer family of proteins, methyltransferases, add the methyl groups to nucleotides whereas reader proteins bind to methylated DNA/RNA and assist in the formation of protein complexes which influence transcriptional or translational processes. The eraser group of proteins are enzymes known as demethylases which generate the oxidised states and takes off methyl groups. The expression of these effector proteins across brain regions and within cellular environments will have important consequences for transcription and translation activity.

To investigate the involvement of 5mC and m^5^C methylation processes contributing to the development of AD neuropathology, we analysed RNA sequencing data from the Aging, Dementia and Traumatic Brain Injury Study to characterise RNA expression profiles of 31 DNA and RNA methylation effector protein genes across four brain regions in 56 unaffected individuals and 51 individuals with AD. In a second phase, we examined gene expression within these DNA/RNA methylation genes by grouping the samples by Braak stage and CERAD specific neuropathological staging. To better understand the relationship between TBI and dementia and changes in effector protein abundance, we also explored gene expression profiles in groups of individuals with the following: no TBI and no AD, all TBI (with and without AD), TBI and no AD, TBI and AD and, AD without TBI.

## Methods

### Study samples

Data were obtained from the Aging, Dementia and Traumatic Brain Injury Study from the Allen Institute for Brain Science (http://aging.brain-map.org/) [34, 35]. The cohort of case and age–matched unaffected control individuals was provided by the Adult Changes in Thought (ACT) Study [36]. Information was gathered from 107 individuals consisting of 51 individuals with clinical diagnosis of AD and 56 individuals without AD (referred to as control individuals). Twenty-six of the control individuals had a history of TBI, and 30 had no history of TBI, whereas 27 individuals with AD had a history of TBI and 24 had no history of TBI (Table 1). Brain tissue was procured by macrodissection from fresh frozen brain with a mean post-mortem interval (PMI) of 4.6 ± 1.5 h for AD individuals and 4.7 ± 2 h for control individuals [34]. RNA expression values were obtained for the hippocampus (HIP), the superior temporal gyrus (STG) of the temporal cortex, the inferior parietal cortex (IPC) and the white matter (WM) from the parietal cortex (Supplementary Table 1).

**Table 1:** Demographic characteristics and neuropathological scores of study individuals

	Controls *N* = 56	AD *N* = 51	Test statistic	*p* value
Age, *x̃* (*Q*_1_–*Q*_3_)	89 (84–92)	89 (86–92)	*U* = 133	0.55
Sex, *n* (%)				
Female	21 (47.7)	23 (52.3)	*χ*^2^ = 0.6	0.43
Male	35 (55.6)	28 (44.4)		
*APOE4* allele, *n* (%)				
Carrier	7 (12.5)	13 (25.5)	*χ*^2^ = 5.3	0.07
No carrier	47 (84.0)	33 (64.7)		
NI	2 (3.5)	5 (9.8)		
CERAD scores, *n* (%)				
0–1	37 (65.0)	20 (35.0)	*χ*^2^ = 7.7	0.005**
2–3	19 (38.0)	31 (62.0)		
Braak stages, *n* (%)				
0–II	22 (39.3)	9 (17.7)	*χ*^2^ = 14.7	0.0006***
III–IV	26 (46.4)	18 (35.3)		
V–VI	8 (14.3)	24 (47.0)		
TBI status				
TBI	26	27	*χ*^2^ = 0.5	0.50
No TBI	30	24		

The demographic characteristics, neuropathological scores and TBI status are presented for all individuals and for individuals within the groups no AD and AD. *x̃*, median; *Q*_1_, lower quartile; *Q*_3_, upper quartile; *U*, Mann–Whitney test; *χ*^2^, chi-square

*APOE4* apolipoprotein E4, *CERAD* Consortium to Establish a Registry for Alzheimer’s Disease, *NI* no information

^**^p < 0.01; ***p < 0.001

Diagnosis criteria for AD established by the National Institute of Ageing and the CERAD [6] is based on the ‘ABC’ score which consists of the Aβ senile plaque rating, a Braak score and a CERAD staging. The Braak score measures the presence and distribution of NFT through specific brain regions and is classified in stages 0 to VI accordingly. The CERAD rating is a semiquantitative assessment of the presence of Aβ as neuritic plaques (Supplementary Table 2).

### Analysis of RNA expression profiles

mRNA expression profiles were examined for the following genes: DNA methylation writers,* DNMT1* (NM_001130823), *DNMT3A* (NM_022552.5) and *DNMT3B* (NM_006892.4); readers, *MeCP2* (NM_004992.4), *ZBTB4* (NM_001128833.2), *ZBTB33* (NM_001184742.2), *ZBTB38* (NM_001376113.1) and *UHRF1* (NM_001048201.3); and erasers, *TET1* (NM_030625.3), *TET2* (NM_001127208.3), *TET3* (NM_001287491.2),* TDG* (NM_003211.6), *MBD4* (NM_001276270.2), *AICDA* (NM_020661.4), *GADD45A* (NM_001924.4), *GADD45B* (NM_015675.4) and *GADD45G* (NM_006705.4). RNA methylation effector genes examined were as follows: writers, *NOP2/NSUN1* (NM_001258308.2), *NSUN2* (NM_017755.6), *NSUN3* (NM_022072.5), *NSUN4* (NM_199044.4), *NSUN5* (NM_148956.4), *NSUN6* (NM_182543.5), *NSUN7* (NM_024677.6) and *TRDMT1/DNMT2* (NM_004412.7); readers, *ALYREF* (NM_005782.4) and *YBX1* (NM_004559.5); and erasers, *ALKBH1* (NM_006020.3), *TET1* (NM_030625.3), *TET2* (NM_001127208.3) and* TET3* (NM_001287491.2). Expression data were obtained from normalised fragments per kilobase of transcript per million (FPKM) values derived from RNA sequencing and presented as a z-score value of expression.

Individuals were categorised by clinical diagnosis AD versus control and grouped by age of death, *APOE4* allele carrier status and Braak and CERAD staging in HIP and STG (Supplementary Table 3) and IPC or WM (Supplementary Table 4). For Braak staging, three groups were generated to represent levels of pathology. Group 1 corresponded to individuals representing low levels of pathology with a Braak stage between 0 and II. Group 2 had a Braak stage between III and IV and had moderate pathology. In the third group, samples had Braak stages between V and VI, indicating the highest level of pathology. To analyse by CERAD ranking, samples were divided into two groups with a CERAD score of 0–1 indicating low amyloid load and a CERAD score of 2–3 representing high amyloid load.

Supplementary Fig. 1 provides a flow chart of our analysis pipeline. In a first phase, differences between age, sex, *APOE4* allele status and Braak and CERAD staging in the groups AD and control were assessed. We then tested for significant correlations between diagnosis status, Braak, CERAD and *APOE4* across each of the four brain regions. In a second phase, we compared RNA abundance profiles across the 32 effector proteins in AD and control groups for each brain region. Subsequently, differences in gene expression were examined in individuals classified by Braak and CERAD rankings. In the third arm of the study, we assessed differential expression in individuals who self-reported TBI. For this analysis, the cohort was divided into five groups: a TBI-control group of aged individuals without TBI and without AD (referred to as ‘TBI-control’); a group of individuals which includes all TBI individuals, i.e. with and without dementia (All TBI); a third group of individuals with TBI but no AD (TBI + no AD); a fourth group which includes individuals with TBI and AD (TBI + AD); and a final group with no TBI but have AD (no TBI + AD). No differences were observed between gender, age and brain tissue post-mortem interval across the TBI groupings (Supplementary Tables 5 and 6). Similarly, in individuals who reported head injuries, we found no difference between age of first TBI incident, number of TBI incidents with loss of consciousness or duration or across the TBI groupings (Supplementary Table 7).

### Statistical analysis

Normality of gene expression z-scores was tested by applying the Shapiro–Wilk test as well as visual analysis of Q-Q plots. Descriptive statistics was performed presenting the mean with standard deviation (SD) or median with 95% confidence interval (CI). In the first stage of analysis, differences in age between diagnosis status were examined using the Mann–Whitney test. Chi-square tests were performed to assess the relationships between diagnosis status and sex, *APOE4* and Braak and CERAD scores. Differences in gene expression values of DNA and RNA effector proteins between AD and controls, and between the CERAD groupings, were evaluated using *t* tests and Mann–Whitney tests. To identify significant differences in the expression values between Braak groups, a one-way ANOVA or Kruskal–Wallis test was performed with the Tukey post hoc test or Dunn’s test analysis, respectively. In the final stage of analysis, corrections for multiple comparisons across TBI groupings were performed using Dunnett’s test for multiple comparisons. All tests were two tailed, and a level of significance of 0.05 was accepted. Data analysis and graphs were generated using IBM SPSS Statistics for Windows (version 27.0) and GraphPad Prism (version 9.1.1) for Windows.

## Results

### Demographics and neuropathologic load in control and Alzheimer’s disease groups

We first examined differences in group demographics and pathology scores between control individuals and individuals with AD (Table 1). No significant differences were observed for age, sex, *APOE4* allele status or TBI history between the groups. We found a significant difference between AD and control individuals in Braak staging (*χ*^2^ = 14.7, *p* = 0.0006) and CERAD scores (*χ*^2^ = 7.7, *p* = 0.005). As expected, AD samples had a higher prevalence of Braak V–VI and of CERAD 2–3 staging, whilst most control individuals presented with a Braak stage of 0–IV and a CERAD score of 0–1. This pattern was also consistently observed across the four brain regions with the highest AD case and control group difference for Braak (*χ*^2^ = 17.2, *p* = 0.0002) and CERAD (*χ*^2^ = 12.4, *p* = 0.0004) ranks in the hippocampus. As expected, a significant positive correlation between Braak and CERAD scores was apparent across all brain regions (*r* = 0.6, *p* < 0.0001) (Supplementary Fig. 2).

### Effector protein expression profiles in control and Alzheimer’s disease individuals

**Fig. 1:**
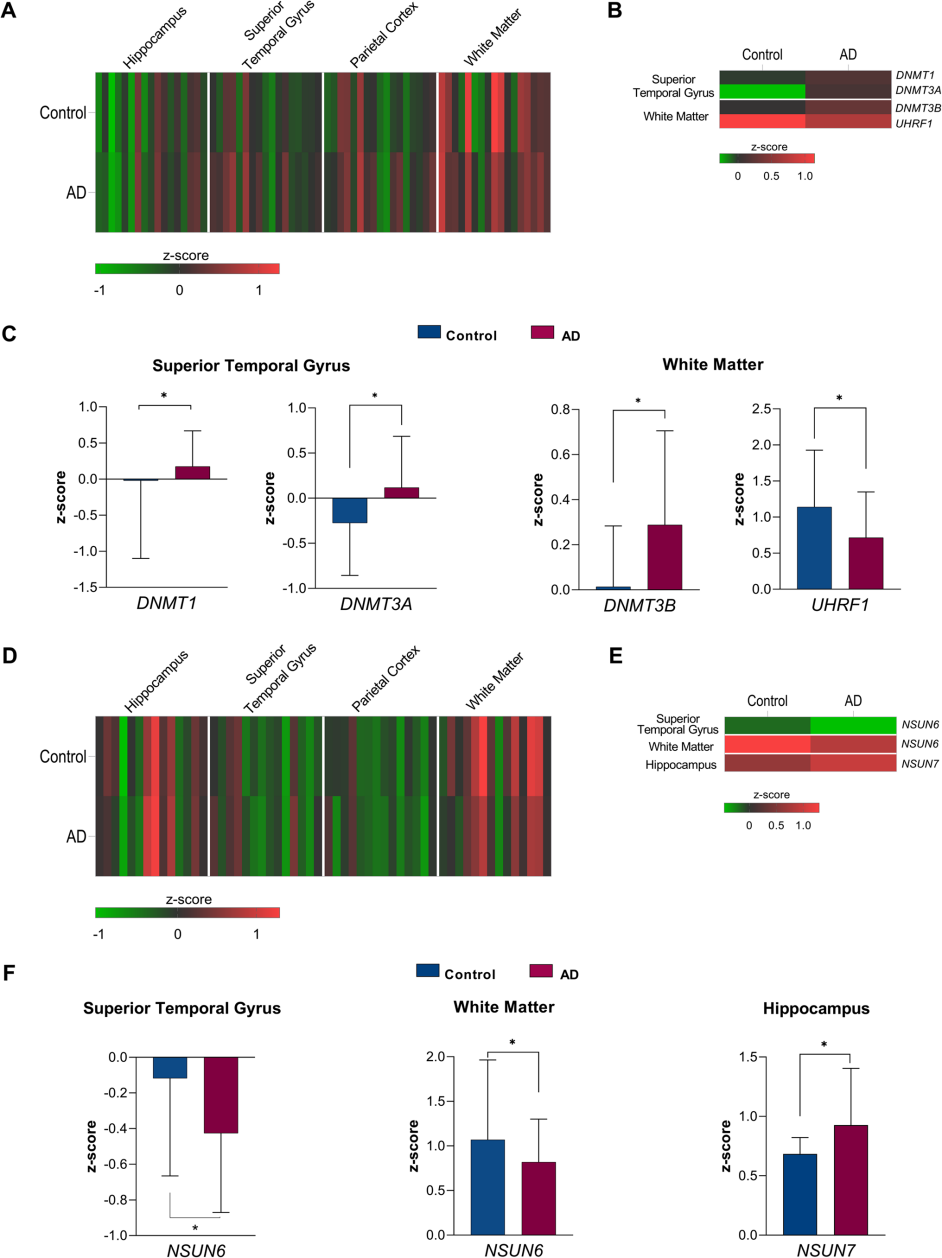
Expression profiles of DNA and RNA effector proteins within the hippocampus, superior temporal gyrus, parietal cortex and white matter tissue. **A** Relative expression of DNA effector proteins in non-affected individuals and individuals with AD. **B**, **C** Significantly increased expression of DNA effector proteins, *DNMT1*, *DNMT3A* and *DNMT3B*, and decreased expression of *UHRF1* were evident in individuals with AD as compared to unaffected individuals in the superior temporal gyrus and white matter regions. **D** Relative expression of RNA effector proteins in non-affected individuals and individuals with AD. **E**, **F** Significantly decreased expression of the RNA writer effector protein *NSUN6* in AD individuals in the white matter and superior gyrus and significantly increased abundance of *NSUN7* in AD individuals in the hippocampus were observed. In **A**, **B**, **D** and **E**, low expression values, < 0, are presented as green and high expression value, > 0, is shown as red. *p < 0.05

To determine patterns of expression across the brain regions in the normal brain, we first examined the expression profiles of effector proteins in control individuals (Fig. 1). In healthy aged individuals, DNA effector proteins showed varied expression across the four brain regions and generally showed higher expression in the WM and lowest expression in the HIP (Fig. 1A, Supplementary Fig. 3A). The STG and parietal cortex (IPC) were found to have similar gene expression patterns and indicated that the writer *DNMT3B*, readers *MeCP2* and *ZBTB4* and erasers *GADD45B* and *GADD45G* had the highest abundance in these regions. In the WM of the parietal lobe, the writers *DNMT1* and *DNMT3A*, the reader *UHRF1* and the erasers *TET1* and *TET2*, which are involved in both DNA and RNA modification, were found to be the most highly expressed transcripts. These observations suggest that individual effector proteins may have varied roles in DNA methylation processes depending on the brain region and that function may be dependent on the cell and tissue context.

In the aged individuals with Alzheimer’s disease, DNA effector proteins commonly showed similar tissue-specific patterns in expression to the non-affected samples but showed overall higher abundance of effector transcript expression in the STG, IPC and WM as compared to control individuals (Fig. 1A and Supplementary Fig. 3A). The eraser proteins indicated the most varied expression profiles across the brain regions and typically showed increased expression in AD cases across all regions. Quantitative comparisons of gene expression from DNA effectors between AD and control individuals (Con) revealed significant differences in expression of the writers *DNMT1* (*p* = 0.01) and *DNMT3A* (*p* < 0.05) in the STG and *DNMT3B* (*p* < 0.05) in the WM (Fig. 1C, Supplementary Table 8). In all significant differences, higher expression was observed in AD cases compared to controls. Conversely, the DNA reader *UHRF1* showed significantly higher abundance in control individuals compared to AD cases (*p* < 0.05) in the WM (Fig. 1B and 1C). No significant differences in 5mC effector protein abundance were observed in the IPC or HIP.

Like the DNA effector proteins, RNA effector proteins in the healthy brain exhibited overall highest relative expression in the WM tissue (Fig. 1D, Supplementary Fig. 3B). However, in contrast to DNA effector proteins, gene expression of effector proteins in the hippocampus, although varied, was moderately high particularly for the writer proteins with the exception of *NSUN4*. *YBX1* and *TET1* and *TET2* were the most abundant reader and eraser proteins, respectively, in the HIP and WM, whereas in the STG and IPC, the reader *ALYREF* and the erasers *ALKBH1* and *TET3* were more highly expressed again providing evidence for tissue-specific RNA methylation effector protein mechanisms.

RNA effector proteins in the AD group again showed a similar overall expression profile to healthy controls across the brain regions (Fig. 1D). However, in the AD group, the writer *NSUN6* was found to have significantly lower abundance in two regions: in the STG (*p* = 0.02) and in the WM (*p* = 0.03), whereas *NSUN7* showed higher abundance in the HIP (*p* = 0.02) (Fig. 1E and 1F, Supplementary Table 8). No differences in the expression of the writers between AD and control individuals were found in the IPC or for any reader or eraser protein transcripts in any of the four brain regions.

### Differences in DNA/RNA effector protein expression grouped by Braak and CERAD neuropathological scales

**Fig. 2:**
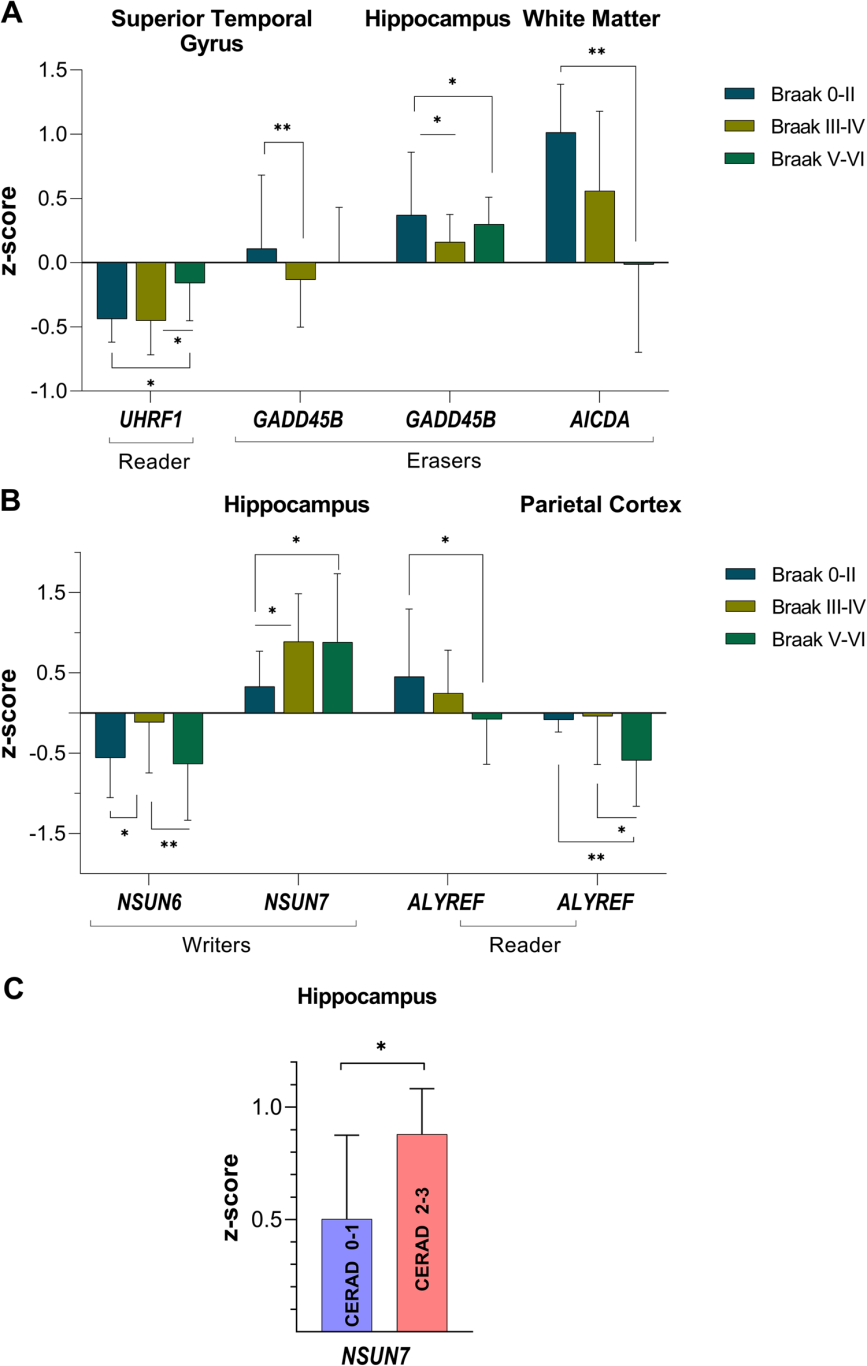
DNA and RNA methylation effector proteins show significant differences in abundance across Braak stages and CREAD rankings. **A** The reader *UHRF1* and eraser *GADD45B* showed lower expression in the mid Braak stages in the superior temporal gyrus. Significantly lower expression of mid-Braak stages was also evident for *GADD45B* in the hippocampus whereas *AICDA* was significantly reduced in tissue with the highest Braak pathology in the white matter. **B** The writers *NSUN6* and *NSUN7* and the reader *ALYREF* showed significant differences in relative expression across Braak stages in the hippocampus and inferior parietal lobe. **C** Consistent with these Braak staging, *NSUN7* was significantly increased in individuals with the highest neuropathological CERAD score in the hippocampus. *p < 0.05; **p < 0.01

Table 2 presents the relative expression values and associated *p* values for differences in 5mC DNA and m^5^C RNA methylation effector transcript expression grouped by Braak stages. We observed significantly higher abundance in the two DNA eraser protein transcripts *GADD45B* and *AICDA* which were associated with differences in Braak staging (Fig. 2A). Here, *GADD45B* gene expression was significantly lower in the mid-neuropathology scores compared to low-early and high-late neuropathological Braak staging in the hippocampus (*H* = 6.9, *p* = 0.03; post hoc between stages 0–II and III–IV, *p* = 0.01) and the superior temporal gyrus (*H* = 7.0, *p* = 0.03; post hoc between stages 0–II and III–IV, *p* = 0.008). Similarly, expression of the eraser *AICDA* in the WM was significantly higher in low-early Braak stages compared to both mid and late stages (*H* = 10.1, *p* < 0.01; post hoc between stages 0–II and V–VI, *p* = 0.002; post hoc between stages III–IV and V–VI, *p* = 0.03). Conversely, and consistent with differences between healthy aged and AD tissues, the reader effector transcript *UHRF1* showed significant lower expression in low and mid Braak staging groups compared to Braak V–VI late stages in the STG (*H* = 7.9, *p* = 0.02; post hoc between stages 0–II and V–VI, *p* = 0.02; post hoc between stages III–IV and V–VI, *p* = 0.01).

**Table 2:** Significant differences in the expression of 5mC DNA and m^5^C RNA methylation effector proteins between Braak stages

	Braak stages							
						Post hoc		
	0–II	III–IV	V–VI	All groups		0–II vs III–IV	0–II vs V–VI	III–IV vs V–VI
	*x̃* (95% CI)			Statistic (*H*)	*p* value	*p* value		
DNA effector proteins								
Hippocampus								
*GADD45B*	0.37 (0.66)	0.16 (0.24)	0.30 (0.53)	6.88	0.03*	0.01*	0.38	0.10
Superior temporal gyrus								
*GADD45B*	0.11 (0.42)	− 0.13 (− 0.02)	0.00 (0.23)	7.01	0.03*	0.008**	0.20	0.21
*UHRF1*	− 0.44 (− 0.36)	− 0.45 (− 0.32)	− 0.16 (0.30)	7.88	0.02*	0.96	0.019*	0.01*
White matter								
*AICDA*	1.01 (1.41)	0.56 (0.91)	− 0.25 (0.50)	10.07	0.006**	0.22	0.002**	0.03*
RNA effector proteins								
Hippocampus								
*NSUN6*	− 0.56^†^ (0.70)	− 0.14^†^ (0.73)	− 0.67^†^ (0.83)	4.67^†^	0.01*	0.03*	0.60	0.005**
*NSUN7*	0.34^†^ (0.70)	0.96^†^ (1.11)	0.96^†^ (1.05)	3.79^†^	0.03*	0.01*	0.02*	0.98
*ALYREF*	0.45 (0.84)	0.25 (0.57)	− 0.08 (0.21)	6.23	0.04*	0.24	0.01*	0.14
Parietal cortex								
*ALYREF*	− 0.09 (0.42)	− 0.04 (0.32)	− 0.59 (-0.29)	8.36	0.02*	0.37	0.006**	0.03*

The median (*x̃*), 95% CI values per Braak group, test statistic and *p* value for comparisons across all groups and per post hoc comparison are presented

^*^p < 0.05; **p < 0.01

^†^Mean, standard deviation and *F* statistic are presented for these values

Differences in levels of expression of the RNA methylation effector transcripts *NSUN6*, *NSUN7* and *ALYREF* were also observed across the Braak staging groups (Table 2, Fig. 2B). In the hippocampus, *NSUN6* showed lower expression in the early and late neuropathological Braak stages I–II and V–VI (*F* = 4.7, *p* = 0.01; post hoc between stages 0–II and III–IV, *p* = 0.03; post hoc between stages III–IV and V–VI, *p* = 0.005). In contrast, *NSUN7* showed higher expression in mid and later Braak stages (*F* = 3.8, *p* = 0.03; post hoc between stages 0–II and III–IV, *p* = 0.01; post hoc between stages 0–II and V–VI, *p* = 0.02). This finding is consistent with a higher relative expression of *NSUN7* in CERAD stages 2–3 compared to CERAD stages 0–1 (Fig.2C) (*p* = 0.03) (CERAD 0–1, *x̃* = 0.50; CERAD 2–3, *x̃* = 0.88). In contrast, *ALYREF* showed significantly low abundance in late Braak stages V–VI in both the hippocampus (*H* = 6.2, *p* = 0.04; post hoc between stages 0–II and V–VI, *p* = 0.01) and the parietal cortex (*H* = 8.4, *p* = 0.02; post hoc between stages 0–II and V–VI, *p* = 0.006; post hoc between stages III–IV and V–VI, *p* = 0.03) (Fig. 2B, Table 2).

**Fig. 3:**
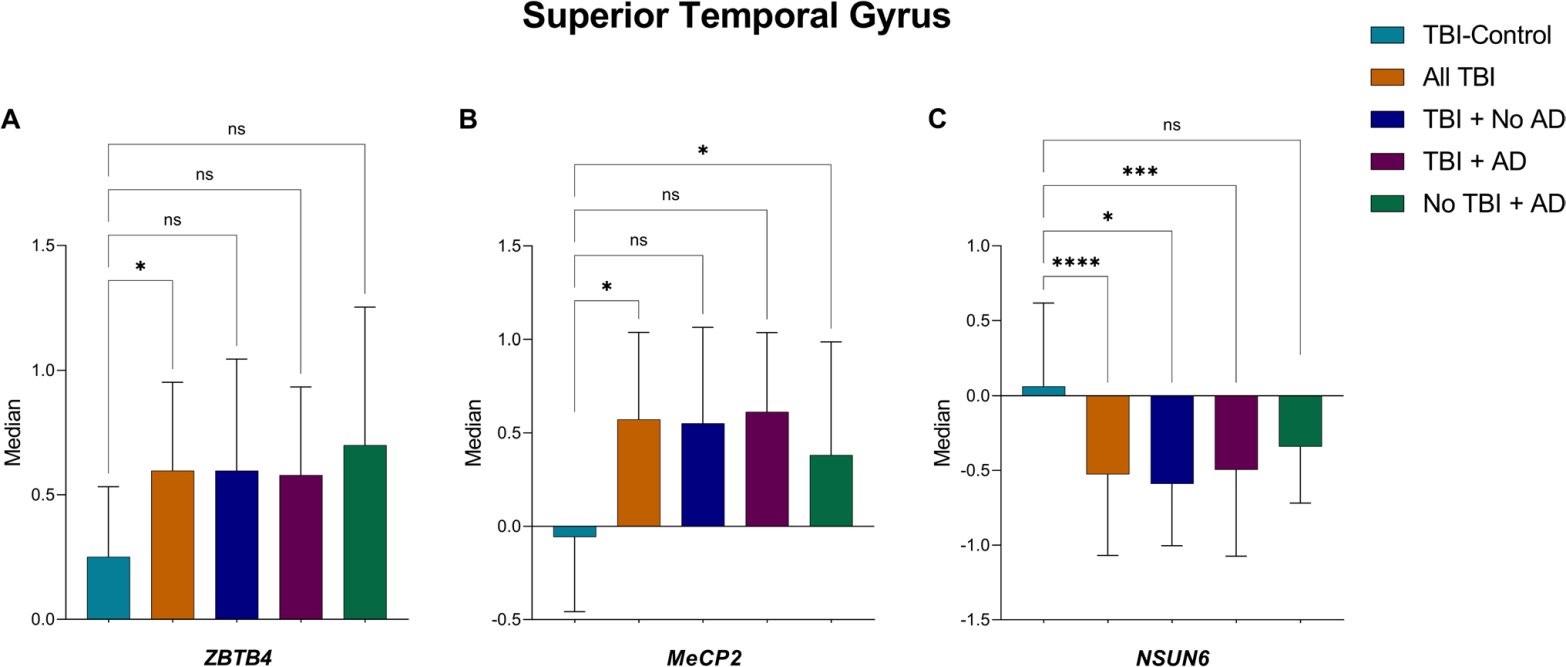
Cytosine methylation effector proteins showing significant differential expression between traumatic brain injury (TBI) groupings. All significant differences in expression between TBO groupings were found in the superior temporal gyrus. **A** The DNA effector reader protein *ZBTB4* showed significant decreased expression in TBI-controls compared with the all-TBI group. **B** The DNA reader *MECP2* showed significantly higher expression in the all-TBI group and no TBI + AD group compared with the control-TBI individuals. **C** The RNA effector writer protein *NSUN6* showed significantly higher expression in the TBI-control group as compared to all individuals with TBI, individuals with TBI and no AD, and TBI and AD. *p ≤ 0.05; ***p ≤ 0.001; ****p ≤ 0.0001

In the final stage of the study, expression of 5mC DNA and m^5^C RNA methylation effector proteins was investigated within the five TBI groupings: TBI-control (no TBI + no AD), all TBI, TBI + no AD, TBI + AD and no TBI + AD (Supplementary Fig. 1). Two DNA readers, *ZBTB4* and *MeCP2*, showed significant changes in RNA abundance, and both were observed to be less abundant in the ‘TBI-control’ group compared to the ‘all TBI’ group (*p* < 0.05) (Fig. 3A and 3B). *MeCP2* was also observed to be less abundant in the TBI-control group when compared to the ‘no TBI + AD’ group (*p* < 0.05) (Fig. 3B, Supplementary Table 9). Finally, similar to the AD and Braak scoring analysis, the RNA methylation writer *NSUN6* exhibited a significant difference across these TBI groupings. In the STG, we observed a significantly lower expression of *NSUN6* in the group ‘TBI + no AD’ (*p* ≤ 0.05) and in groups ‘all TBI’ (*p* ≤ 0.0001) and ‘TBI + AD’ (*p* ≤ 0.001), when compared to the TBI-control group (Fig. 3C, Supplementary Table 9). These findings suggest that decreased expression of *NSUN6* in TBI is not driven by a dementia phenotype.

## Discussion

Epigenetic processes have become increasingly relevant in understanding disease-modifying mechanisms [37–39]. In the current study, we observed changes in the expression of the DNA writer proteins *DNMT1*, *DNMT3A* and *DNMT3B* and of the reader *UHRF1* in AD individuals whilst the DNA methyltransferase erasers *GADD45B* and *AICDA* showed changes in mRNA abundance within neuropathological load groupings. RNA methylation writers *NSUN6* and *NSUN7* presented contrasting profiles, with *NSUN6* significantly decreased in AD individuals and individuals with TBI in the hippocampus and temporal gyrus, whereas *NSUN7* was increased within the hippocampus in individuals with AD or high neuropathology. *ALYREF*, a RNA reader, showed decreased abundance with higher pathological stages, and a history of TBI was associated with significant increases in DNA readers *ZBTB4* and *MeCP2*, but a decrease in *NSUN6* abundance.

DNMT writer proteins interact with chromatin and histone modification enzymes to stabilise the methylation machinery complex and direct the addition of methyl groups onto DNA [40]. DNA reader proteins bind to methylated DNA CpH sites and repress or, in the case of MeCP2, potentially activate transcription [41]. The functional consequences of mutations within *DNMT1*, *DNMT3B* and *DNMT3A*, which cause monogenic human diseases and rare cancers, e.g. *DNMT1* complex disorders (HSAN1E) [42, 43], *DNMT3B* (ICF syndrome) [44, 45] and *DNMT3A* (Tatton Brown Rahman syndrome) [46, 47], are proposed to be dependent on mutation type and the extent they cause a reduction in gene function or expression, i.e. the degree of hypomorphism. As a result, DNMT complex activity can be either weakly, moderately or strongly disrupted and can cause localised changes or more global widespread changes in DNA methylation [48–51]. Therefore, our findings of differential expression of *DNMT1*, *DNMT3*, *DNMT3B* and *UHRF1* in AD and *ZBTB4* and *MeCP2* in TBI could lead to alterations in complex binding which is consistent with causing changes to DNA methylation sites. Indeed, evidence already exists that disruption of MeCP2 in neuronal tissue in mice increases expression of long neuronal genes through binding to mCA and mCG within, and neighbouring, long neuronal genes [52, 53]. However, whether changes to the writer and reader complexes commonly affect widespread or specific patterns of unoxidised and oxidised mCG and mCA cytosine bases which influence increased transcriptional activity remain to be investigated.

RNA methylation effector proteins are known to target various types of RNA species. For example, m^5^C occurs on mRNA, tRNA, rRNA and mt-RNA and alternative non-coding RNA species such as enhancer RNA (eRNA), vault RNA (vtRNA) and circular RNA (circRNA) [54–56]. Consequently, disruption to methylated bases could have multiple effects on cellular processes. We found consistent and contrasting differences in increases and decreases in expression in two m^5^C RNA writer proteins, *NSUN7* and *NSUN6*, within the hippocampus and with disease/pathology groups. The function of NSUN7 is still relatively unknown although it has been suggested to regulate the stability of enhancer RNAs of genes targeted by the transcriptional co-activator PGC-1α [57]. Interestingly, PGC-1α, a known mitochondria and energy metabolism regulator, has been linked to neuronal survival and synaptic maintenance and its dysregulation is suggested to be involved in pathogenesis of neurodegenerative diseases [58–60].

In contrast, NSUN6 is known to methylate tRNA^Thr^ and tRNA^Cys^ tRNA molecules in human cells [61] and was recently discovered, together with NSUN2, to determine most mRNA transcription-wide m^5^C sites [62]. Furthermore, NSUN6 is associated with what has been termed ‘type II’ m^5^C mRNA sites which contain a downstream UCCA motif and are predicted to be located in the loops of putative hairpin structures [62–64]. Like NSUN6, type II m^5^C mRNAs are commonly found in the cytoplasm of cells and in NSUN6 knock-out cells, and type II modification is associated with a modest overall increase in translation efficiency although translational efficiency is dependent on genic location [62]. Of interest, within several regions of the human brain, a circular RNA transcript is predicted to be encoded within *NSUN6* coding regions [65, 66]. As circular RNAs can regulate mRNA through binding to RNA binding proteins such as RNA methylation effector proteins and have been shown to be abnormally expressed in Alzheimer’s disease brain [67], this highlights the potential for a more complex transcriptional regulatory system involving *NSUN6* underlying AD pathology.

The m^5^C reader ALYREF, also known as THOC4, also showed increased expression with increasing Braak pathology. ALYREF has been described as the main regulator of m^5^C-modified mRNA export out of the nucleus, and mutations within transcription and export (TREX) complex proteins involved in the export process cause syndromic forms of intellectual disabilities [68–70]. Moreover, the recognition and hence nuclear-cytoplasmic shuttling of specific transcripts by ALYREF is reduced with the knockdown of NSUN2 [30, 71]. Together, these observations suggest that both NSUN6 and ALYREF require NSUN2 for their function in the distribution of m^5^C mRNA sites as well as of m^5^C mRNA transport into the cytoplasm and that tissue-specific changes in m^5^C writer abundance may have cell type–dependent consequences on protein translation and protein complexes within cytoplasmic sites.

Many studies have reported differential 5mC DNA methylation at mCG bases in AD or with Braak stage neuropathology. However, these reports mostly come from candidate gene studies or methylation array studies which examine pre-selected, known CpG sites often preferentially located within promoter regions [72–74]. To date, very few studies have performed hypothesis-free bisulphite DNA sequencing and hence have comprehensively examined, at a base resolution, mCG/mCH or 5hmC sites in individuals with dementia or TBI [75, 76]. As yet, no studies have assessed single-base transcriptome-wide mRNA m^5^C methylation in cohorts with neurodegenerative diseases. Therefore, the consequences of change to the DNA and RNA methylome and relationship to pathological processes are still unclear. However, the non-protein α-amino acid homocysteine is an important intermediate in the one-carbon pathway which is essential for the production of methyl groups available for DNA/RNA methylation [77]. High homocysteine is also an established risk factor for both AD and TBI [39, 78–80] and has been suggested to increase β-amyloid and tau pathology, protein aggregation as well as mitochondrial dysfunction involving oxidative stress pathways [81–84]. Further studies are needed to elucidate the functional relationship between changes in homocysteine, methylation effector-protein processes and pathology-inducing mechanisms.

One limitation of this study is that changes in expression were identified from heterogenous cellular tissue sections and were therefore not cell-type population specific or subcellular region specific. Cell nuclei extracts from healthy human brain tissue have been reported to show cell population–specific differences in 5mC DNA profiles with differences between oligodendrocyte and neuron population profiles estimated to be as high as ~ 35% [85]. Alterations in cell type composition in brain tissue with increased pathological staging are also well documented, e.g. atrophy of neuronal and glial cell populations concurrent with increases in reactive astrocytic and microglial population abundance. Such shifts in cell type abundance are one explanation for age-associated, or Braak stage–associated, changes in 5mC DNA [86, 87] or m^5^C RNA methylation profiles, and which could be a significant factor influencing effector protein transcript expression. Alternatively, mRNA methylation effector proteins are known to undergo autoregulation, i.e. are modified themselves [88], which potentially influences their spatiotemporal transcriptional abundance and hence regulatory feedback loops.

A second related limitation of this study is that we cannot say whether changes in effector transcript expression result in changes in protein abundance and hence whether post-transcriptional regulatory mechanisms, proteomic changes or a combination of both mechanisms may be influencing pathological processes. Indeed, knockdown of mouse *Nsun2*, which, like NSUN6, is part of the tRNA regulome, results in decreased tRNA m^5^C levels, deficits in tRNA glycine codon–specific defects and a loss of Gly-rich synaptic proteins [89]. Whether such consequential molecular changes are co-moderated by *Nsun2* effector transcript mechanisms remains to be determined. However, cell population–specific high-throughput transcriptomic and proteomic studies of dementia and TBI pathological brain tissue will be necessary to elucidate methylome-specific mechanisms and consequences.

Our findings provide novel evidence of epitranscriptional control involved in AD and TBI, and with pharmacological targeting of DNA and RNA methyltransferases and methylation pathways currently underway for forms of cancers, new therapeutic avenues for dementia may advance.

## Supplementary Information

Below is the link to the electronic supplementary material.

Supplementary file1 (DOCX 626 KB)

## Data Availability

The dataset analysed during the current study are publicly available from the Allen Institute for Brain Science, University of Washington Medicine, and Kaiser Permanente Washington Health Research Institute (2016) (Aging, Dementia, and TBI Study [NG00059], available from aging.brain-map.org. RRID:SCR_014554 | Primary publication: Miller J. A., et al. (2017). Neuropathological and transcriptomic characteristics of the aged brain. eLife, 2017;6:e31126. https://doi.org/10.7554/eLife.31126).
