# Supplementary material for: Differential expression of m5C RNA methyltransferase genes NSUN6 and NSUN7 in Alzheimer’s disease and traumatic brain injury
Source: Mol Neurobiol. 2023 Jan 17;60(4):2223–35. doi: 10.1007/s12035-022-03195-6 (PMC9984329; doi:10.1007/s12035-022-03195-6)
Supplement: Supplementary file 1 — Supplementary file1 (DOCX 626 KB) [file 12035_2022_3195_MOESM1_ESM.docx]

**Article title**: Differential expression of the m^5^C RNA methyltransferase genes *NSUN6 and NSUN7* in Alzheimer’s disease and Traumatic Brain Injury

**Journal Name**: Molecular Neurobiology

Adriana PerezGrovas-Saltijeral^1^, Anto P. Rajkumar^2,3^, Helen Miranda Knight^1^*

^1^Division of Cells, Organisms and Molecular Genetics, School of Life Sciences, University of Nottingham, Nottingham, UK.

^2^Institute of Mental Health, Mental health and clinical neurosciences academic unit, School of Medicine, University of Nottingham, Nottingham, UK.

^3^Mental health services for older people, Nottinghamshire health care NHS foundation trust, Nottingham, UK.

*Corresponding author: [Helen.knight@nottingham.ac.uk](mailto:Helen.knight@nottingham.ac.uk)

|  |  | **Control**  **n = 56** | **AD**  **n = 51** |
| --- | --- | --- | --- |
| **Brain region** | Hippocampus | 51 | 43 |
|  | Superior temporal gyrus | 50 | 49 |
|  | Inferior parietal cortex | 46 | 45 |
|  | White matter (parietal lobe) | 47 | 46 |

**Supplementary Table 1**. Number of samples in each diagnostic group according to brain region assessed. AD, Alzheimer’s disease.

| **Braak staging for NFT** | |
| --- | --- |
| 0 | No NFT |
| I-II | NFT in entorhinal cortex |
| III-IV | NFT in hippocampus and amygdala |
| V-VI | NFT in neocortex, involving primary motor and sensory areas |
| **CERAD score for density of neuritic plaques** | |
| 0 | No neuritic plaques |
| 1 | Sparse |
| 2 | Moderate |
| 3 | Frequent |

**Supplementary Table 2.** Braak and CERAD scores based on neuropathological criteria for diagnosis of neurodegenerative disease. Information taken from NIA-AA [6].

| **Region** | **Variable** |  | **Total** | **Control** | **AD** |
| --- | --- | --- | --- | --- | --- |
| **Hippocampus** | **# Individuals** | n | 94 | 51 | 43 |
|  | **Braak staging** | 0-II | 27 | 21 | 6 |
|  |  | III-IV | 38 | 23 | 15 |
|  |  | V-VI | 29 | 7 | 22 |
|  | **CERAD** | 0-1 | 47 | 34 | 13 |
|  |  | 2-3 | 47 | 17 | 30 |
|  | **TBI** | Yes | 44 | 22 | 22 |
|  |  | No | 50 | 29 | 21 |
|  | ***APOE4***  **carrier status** | Yes | 71 | 44 | 27 |
|  |  | No | 16 | 5 | 11 |
|  |  | NI | 7 | 2 | 5 |
|  | **Age** | 70-89 | 45 | 25 | 20 |
|  |  | 90-99 | 42 | 25 | 17 |
|  |  | >100 | 7 | 1 | 6 |
| **Superior Temporal gyrus** | **# Individuals** | n | 99 | 50 | 49 |
|  | **Braak staging** | 0-II | 27 | 18 | 9 |
|  |  | III-IV | 43 | 25 | 18 |
|  |  | V-VI | 29 | 7 | 22 |
|  | **CERAD** | 0-1 | 50 | 31 | 19 |
|  |  | 2-3 | 49 | 19 | 30 |
|  | **TBI** | Yes | 48 | 22 | 26 |
|  |  | No | 51 | 28 | 23 |
|  | ***APOE4***  **carrier status** | Yes | 19 | 7 | 12 |
|  |  | No | 74 | 41 | 33 |
|  |  | NI | 6 | 2 | 4 |
|  | **Age** | 70-89 | 53 | 26 | 27 |
|  |  | 90-99 | 40 | 24 | 16 |
|  |  | >100 | 6 | 0 | 6 |

**Supplementary Table 3**. Demographics, APOE4 genotype, neuropathological staging and TBI status of individuals with RNA expression data in the hippocampus and superior temporal gyrus. Data are presented as total number of samples (n). APOE, Apolipoprotein E; NI, No Information; TBI, Traumatic Brain Injury.

| **Region** | **Variable** |  | **Total** | **Control** | **AD** |
| --- | --- | --- | --- | --- | --- |
| **Inferior parietal cortex** | **# Individuals** | n | 91 | 46 | 45 |
|  | **Braak staging** | 0-II | 23 | 15 | 8 |
|  |  | III-IV | 39 | 23 | 16 |
|  |  | V-VI | 29 | 8 | 21 |
|  | **CERAD** | 0-1 | 45 | 28 | 17 |
|  |  | 2-3 | 46 | 18 | 28 |
|  | **TBI** | Yes | 43 | 20 | 23 |
|  |  | No | 48 | 27 | 21 |
|  | ***APOE4***  **carrier status** | Yes | 18 | 7 | 11 |
|  |  | No | 67 | 37 | 30 |
|  |  | NI | 6 | 2 | 4 |
|  | **Age** | 70-89 | 49 | 25 | 24 |
|  |  | 90-99 | 36 | 21 | 15 |
|  |  | > 100 | 6 | 0 | 6 |
| **White matter/ parietal lobe** | **# Individuals** | n | 93 | 47 | 46 |
|  | **Braak staging** | 0-II | 23 | 15 | 8 |
|  |  | III-IV | 40 | 24 | 16 |
|  |  | V-VI | 30 | 8 | 22 |
|  | **CERAD** | 0-1 | 46 | 29 | 17 |
|  |  | 2-3 | 47 | 18 | 29 |
|  | **TBI** | Yes | 44 | 21 | 23 |
|  |  | No | 49 | 27 | 22 |
|  | ***APOE4***  **carrier status** | Yes | 18 | 7 | 11 |
|  |  | No | 69 | 38 | 31 |
|  |  | NI | 6 | 2 | 4 |
|  | **Age** | 70-89 | 50 | 25 | 25 |
|  |  | 90-99 | 37 | 22 | 15 |
|  |  | > 100 | 6 | 0 | 6 |

**Supplementary Table 4**. Demographics, APOE4 genotype, neuropathological staging and TBI status of individuals with RNA expression data in the inferior parietal lobe and white matter of the parietal lobe. Data are presented as total number of samples (n). APOE, Apolipoprotein E; NI, No Information; TBI, Traumatic Brain Injury.


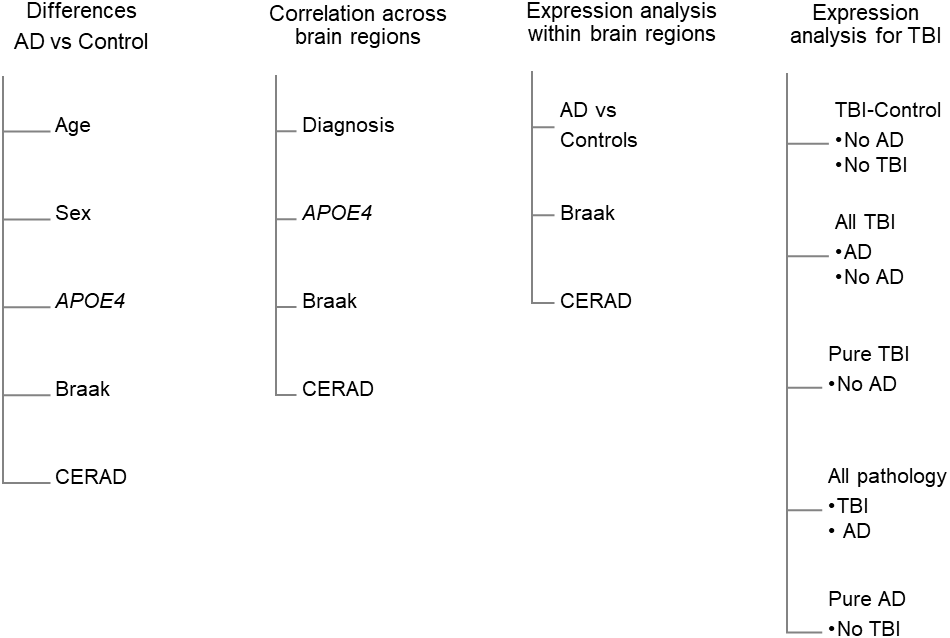

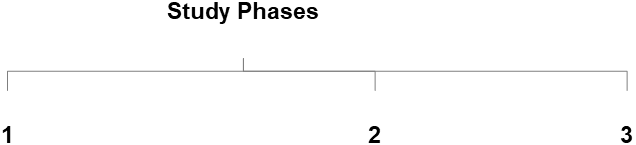


**Supplementary Figure 1**. **Flowchart of the study phases.** The first phase compared individuals with Alzheimer Disease (AD) with healthy individuals and considered the variables age, sex, *APOE4* allele status as well as Braak and CERAD scoring measures. Subsequently, the relationships between diagnosis, *APOE4* and Braak and CERAD scoring were assessed across the four brain regions (STG, HIP, IPC, WM). In a second phase, differences in RNA abundance of the 31 effector proteins were examined first in AD and control individuals, followed by differences by scores of pathological categories Braak and CERAD. In the third phase, individuals were grouped according to TBI and AD status, and expression profiles were examined. AD, Alzheimer’s disease; APOE, Apolipoprotein E; STG, superior temporal gyrus; HIP, hippocampus; IPC, inferior parietal cortex; WM, white matter of the parietal lobe; TBI, traumatic brain injury.

|  | **TBI Cases** | **TBI-Controls** |
| --- | --- | --- |
| **Number of cases** | 53 | 54 |
| **Sex** | 31 males / 22 females | 32 males / 22 females |
| **Age** | 89 +/- 6.3 | 89 +/- 6.2 |
| **PMI (hrs)** | 4.6 +/- 1.5 | 4.7 +/- 2.0 |

**Supplementary Table 5.** Demographic characteristics of TBI cases and TBI-controls. TBI-controls refer to samples with No TBI and No AD. TBI, Traumatic Brain Injury; PMI, Post-mortem interval; AD, Alzheimer’s Disease.

|  | **TBI + No AD** | **TBI + AD** |
| --- | --- | --- |
| **Number of cases** | 27 | 26 |
| **Sex** | 20 males / 7 females | 11 males / 15 females |
| **Age** |  |  |
| **75 – 79**  **80 – 89**  **>90** | 4  10  13 | 2  12  12 |
|  |  |  |

**Supplementary Table 6.** Gender and age of TBI cases subgroups. Individuals were grouped into TBI and no AD or TBI and AD. TBI, Traumatic Brain Injury; AD, Alzheimer’s Disease.

|  | All TBI cases | TBI + No AD | TBI + AD |
| --- | --- | --- | --- |
| Duration of TBI loss of consciousness |  |  |  |
| <10 sec | 36 | 21 | 15 |
| 10 sec – 1 min | 4 | 0 | 4 |
| 1 – 2 min | 9 | 6 | 4 |
| 3 – 5 min | 6 | 4 | 2 |
| 6 – 9 min | 2 | 2 | 0 |
| 10 min – 1 hr | 15 | 8 | 8 |
| >1 hr | 15 | 8 | 8 |
| unknown | 13 | 4 | 9 |
| Number of TBI incidents with loss of consciousness |  |  |  |
| 1 | 81 | 40 | 42 |
| 2 | 15 | 9 | 6 |
| 3 | 4 | 2 | 2 |
| Age at first TBI |  |  |  |
| <10 | 8 | 2 | 6 |
| 10 – 17 | 25 | 13 | 11 |
| 18 – 29 | 23 | 13 | 9 |
| 30 – 64 | 11 | 4 | 8 |
| >65 | 34 | 19 | 15 |

**Supplementary Table 7.** Duration of TBI loss of consciousness, number of TBI with loss of consciousness and age of first TBI occurrence in individuals with head injuries. TBI cases are grouped as all TBI individuals, TBI with no diagnosis of AD, and TBI with a diagnosis of AD. TBI, Traumatic Brain Injury; AD, Alzheimer’s Disease.


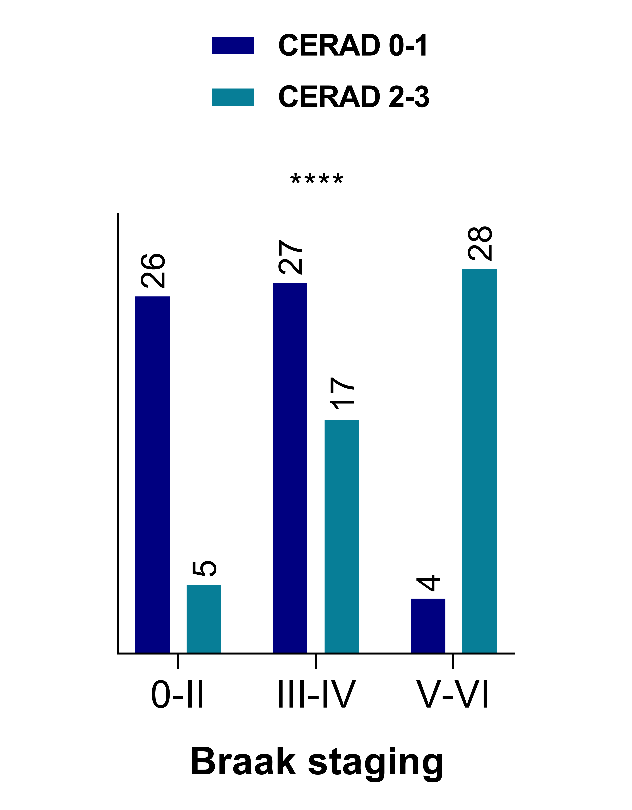


**Supplementary Figure 2.** Correlation between histopathological diagnostic scales Braak and CERAD, across the 4 brain regions. r = 0.616.****p < 0.0001; r, Spearman r.

**
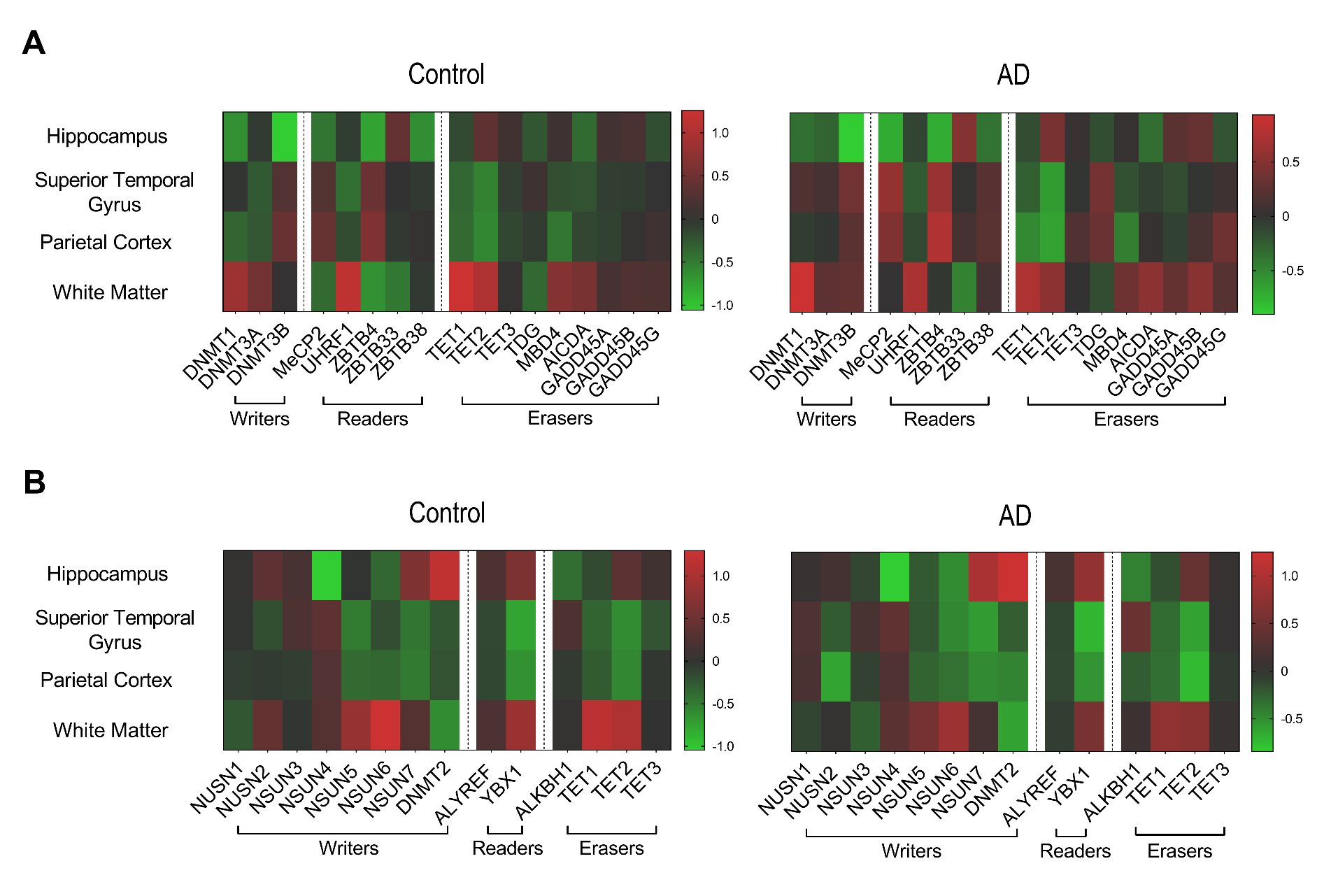
**

**Supplementary Figure 3.** Expression profiles of DNA and RNA effector proteins within the hippocampus, superior temporal gyrus, parietal cortex, and white matter tissue. **(A)** Relative expression of DNA writers, readers and erasers in non-affected individuals and individuals with AD. **(B)** Relative expression of RNA writers, readers and erasers in non-affected individuals and individuals with AD. Values of expression are shown as Z-scores, were < 0 represents low expression in green and high expression values of > 0 are presented in red.

|  |  |  | **Control** | | AD |  |  |
| --- | --- | --- | --- | --- | --- | --- | --- |
| **Tissue** | **Effectors** |  | ***x̃ (Q_1_ - Q_3_)*** | | | ***P* values** | |
| DNA | |  |  | |  |  |  |
| STG | *DNMT1* |  | -0.02  (-1.10 - 0.28) | | 0.17  (-0.31 - 0.67) | 0.010* |  |
|  | *DNMT3A* |  | -0.28  (-0.86 - 2.30) | | 0.12  (-0.43 - 0.69) | 0.046* |  |
|  |  |  |  | |  |  |  |
| WM | *DNMT3B* |  | 0.01  (-0.61 - 0.29) | | 0.29  (-0.12 - 0.71) | 0.013* |  |
|  | *UHRF1* |  | 1.14  (0.61 - 1.93) | | 0.72  (0.11 - 1.35) | 0.038* |  |
| RNA | |  |  | |  |  |  |
| HIP | *NSUN7* |  | 0.68  (0.14 - 0.93) | | 0.93  (0.41 - 1.60) | 0.023* |  |
|  |  |  |  |  | |  |  |
| STG | *NSUN6* |  | -0.12  (-0.67 - 0.44) | | -0.42  (-0.87 - -0.17) | 0.022* |  |
|  |  |  |  | |  |  |  |
| WM | *NSUN6* |  | 1.07  (0.45 - 1.96) | | 0.82  (0.18 - 1.30) | 0.031* |  |
|  |  |  |  | |  |  |  |

**Supplementary Table 8.** **RNA expression values of DNA and RNA methylation effector proteins in control and AD individuals.** STG, Superior temporal gyrus; WM, White matter; HIP, Hippocampus; *x̃,* median*; Q1 - Q3,* first and third quartile; *p < 0.05

|  |  | | **TBI-Control (I)** | | **All TBI**  **(II)** | **TBI + No AD (III)** | | | | **TBI + AD (IV)** | **No TBI + AD (V)** |  | **I vs II** | **I vs III** | **I vs IV** | **II vs V** |
| --- | --- | --- | --- | --- | --- | --- | --- | --- | --- | --- | --- | --- | --- | --- | --- | --- |
| **Tissue** | | | ***x̃ (Q_1_ - Q_3_)*** | | | | | | | |  |  | ***P Values*** | | | |
| **DNA effectors** | | |  | | | | | | | |  |  | |  |  |  |
| **STG** | | ***MeCP2*** | -0.06  (-0.46 - 0.54) | 0.57  (0.04 – 1.04) | | | 0.55  (0.05 – 1.06) | 0.61  (-0.15 – 1.06) | | | 0.38  (-0.04 - 0.99) |  | **0.04*** | 0.17 | 0.07 | **0.04*** |
|  | | ***ZBTB4*** | 0.25  (-0.29 - 0.53) | 0.59  (0.33 - 0.95) | | | 0.59  (0.36 – 1.05) | 0.58  (0.12 – 0.93) | | | 0.69  (0.25 – 1.25) |  | **0.04*** | 0.07 | 0.20 | 0.09 |
| **RNA effectors** | | |  | | | | | | | |  |  | |  |  |  |
| **STG** | | ***NSUN6*** | 0.06  (-0.24 - 0.62) | | -0.53  (-1.0- -0.17) | -0.59  (-1.0 - -0.05) | | | -0.49  (-1.01 - -0.29) | | -0.34  (-0.72 - 0.18) |  | **0.0001**  ******** | **0.01*** | **0.0003 ***** | 0.17 |
|  | |  |  | |  |  | | |  | |  |  |  |  |  |  |

**Supplementary Table 9.** **RNA expression values of DNA and RNA methylation effector proteins in individuals with and without Traumatic Brain Injury.** Median and quartiles values and level for significance for DNA and RNA methylation effector proteins which showed significant differences between groups. Groups were categorised according to the individual’s history of TBI (positive or negative) and clinical diagnosis (AD, no AD). TBI-Control (No TBI + No AD TBI). TBI, Traumatic Brain Injury; AD, Alzheimer’s Disease, STG, Superior temporal gyrus; *x̃,* median*; Q1- Q3,* first and third quartile; *p ≤ 0.05, **p ≤ 0.01, ***p ≤ 0.001, ****p ≤ 0.0001.
